# Mechanical Properties of Long Leaves: Experiment and Theory

**DOI:** 10.1007/s10441-020-09397-6

**Published:** 2020-10-31

**Authors:** A. Jakubska-Busse, M. W. Janowicz, L. Ochnio, B. Jackowska-Zduniak, J. M. A. Ashbourn

**Affiliations:** 1 Department of Botany, Institute of Environmental Biology, University of Wrocław, Kanonia 6/8, 50-328 Wrocław, Poland; 2 Faculty of Applications of Informatics and Mathematics, Department of Applied Mathematics, Warsaw University of Life Sciences-SGGW, ul. Ciszewskiego 8, 02-786 Warsaw, Poland; 3 Faculty of Applications of Informatics and Mathematics, Department of Econometrics and Statistics, Warsaw University of Life Sciences-SGGW, ul. Nowoursynowska 159, 02-776 Warsaw, Poland; 4 Faculty of Applications of Informatics and Mathematics, Department of Computer Science, Warsaw University of Life Sciences - SGGW, ul. Nowoursynowska 159, 02-776 Warsaw, Poland; 5 Department of Engineering Science, University of Oxford, Parks Road, Oxford, OX1 3PJ UK

**Keywords:** Long leaves, Elasticity, Cosserat rods, *Epipactis*, Orchids

## Abstract

The static properties of leaves with parallel venation from terrestrial orchids of the genus *Epipactis* were modelled as coupled elastic rods using the geometrically exact Cosserat theory and the resulting boundary-value problem was solved numerically using a method from Shampine, Muir and Xu. The response of the leaf structure to the applied force was obtained from preliminary measurements. These measurements allowed the Young’s modulus of the *Epipactis* leaves to be determined. The appearance of wrinkles and undulation characteristics for some leaves has been attributed to the small torsional stiffness of the leaf edges.

## Introduction

The modelling of plant organs remains an open problem due to the complexity of plant architecture, regardless of the particular organ or any particular taxonomical group of plants being considered. This study focusses on leaves with parallel venation, namely, leaves with a relatively simple structure of veins which can nonetheless exhibit interesting features such as wrinkles and undulation. Leaf undulation is an interesting and relatively well-studied phenomenon observed in the monocotyledons including orchids. It has been found that the waves in the leaf blades in monocots usually appear perpendicular to the leaf length, which demonstrates that as the leaf surface grows it changes correspondingly lengthwise to the leaf blade. It is worth noting that although undulation normally occurs perpendicular to the leaf length, in the initial stages it occurs alongside its length. Displacements in the wrinkled leaf also occur across the leaf blade and whichever way these appear, they demonstrate that the pace of their growth is irregular since wrinkles in a leaf can be of different lengths. Hejnowicz (1992) found that spatial and temporal fluctuations in the pH of the epidermal cell walls aided the undulation. In a study by Jakubska-Busse and Gola (2014) it was shown that the leaf undulation in some orchids does not have any diagnostic value as an unprogrammed intrinsic feature and should not be applied to taxa identification. This paper is a further attempt to identify which characteristics in the mathematical model of such leaves are responsible for this undulation.

The building of mathematical and numerical models to effectively reflect the complex structure of a plant is very complicated. In particular, this requires different morphological and anatomical constructions of individual plant organs and in addition with the appearance of other phenomena on leaf surfaces including the local undulation of leaf blades, leaves frequently have a characteristic rippling pattern at their edges (Marder 2003).

Once it becomes visible, the local undulation of leaf blades in monocots comprises ripples perpendicular to the direction of the longitudinal expansion of the leaf blade (Zagórska-Marek and Wiss 2003). Detailed studies of the mechanism for undulations in leaves have so far been carried out by Hejnowicz (1992) on the garden tulip (*Tulipa gesneriana*) as well as by Liang and Mahadevan (2009) on the plantain lily (*Hosta lancifolia*).

Recently, several interesting studies have been published in which both the shapes and mechanical properties of plants have been investigated both experimentally and analytically. In particular, in Zhao (2015) the chiral growth of organs in aquatic macrophytes has been studied. As a kind of follow-up, Zhao, Liu and Feng have investigated the aeroelastic behavior of *Typha* (an emergent aquatic macrophyte) blades in wind (Zhao et al. 2016). The biomechanical morphogenesis of the leaves and stalks of representative emergent plants, which can stand upright and survive in harsh water environments, has been considered in Zhao et al. (2018). In Zhao et al. (2020), it has been demonstrated that the leaves and stalks of several species of emergent plants exhibit morphologies of twisting and gradient chirality. The static bending and vibrational properties of these plant organs have been investigated. By modelling the leaves and stalks as pre-twisted cantilever beams, the effects of the cross-sectional geometry, loading condition, handedness perversion, twisting configuration, and morphological gradient on their mechanical behavior have been evaluated.

## The Main Model

In Jakubska-Busse et al. (2016), the model of a leaf with the parallel venation considered as a system of coupled elastic beams was developed using the theory of non-linear bending described by Landau and Lifshitz (1993). However, this theory has some important drawbacks which include the fact that the elongation of each beam can only happen due to bending. More importantly, its discussion of the so-called constitutive relations is lacking in detail. In this paper a model of a leaf with the parallel venation based on the Cosserat theory of rods is provided and the following assumptions are made: (i) The shape of the leaf is determined by the distribution of its veins, (ii) Each vein, together with its surrounding tissue, can be represented by a special Cosserat rod (defined below), (iii) The veins are elastically coupled to their nearest neighbours due to the presence of the tissue between the veins, and (iv) Only the main (“first-order”) veins are taken into account explicitly whilst the presence of secondary veins may lead to additional concentrated forces acting upon the principal veins. As a result we aim to show that the characteristic undulation near the edges of a leaf can be the result of the inhomogeneity of constitutive relations along the veins, which is in contrast to the hypothesis in previous research where the undulation was considered to be the result of dislocations in the regions between the veins and in both primary and secondary veins.

### Special Cosserat Rods

In the discussion below the methods of Antman (2006), Rubin (2000), Cao et al. (2006), Cao and Tucker (2008), and Champneys et al. (1997) are followed. The books by Antman and Rubin contain the general theory of elastic structures in terms of the geometrically exact methodology of Cosserats. The paper by Cao deals with single thin rods with mostly engineering applications in mind. In the work by Champneys et al., both experimental and theoretical work on rubber rods (in particular related to their buckling) was performed. From the above studies only the general formulation (which is quite universal) has been followed using the constitutive relations in the form provided by Champneys et al., as it seems particularly convenient.

We let three vectors $\mathbf{e}_1$, $\mathbf{e}_2$, $\mathbf{e}_3$ form a fixed right-handed orthogonal basis.

Let us first consider a *single* rod. Elements of the rod are labelled in terms of the arc-length coordinate *s*, $0 \le s \le L$. We let $\mathbf{r}(s)$ be the position vector of the centre line of the rod with respect to the fixed basis $\mathbf{e}_1$, $\mathbf{e}_2$, $\mathbf{e}_3$. The configuration of the rod in the deformed state is defined by $\mathbf{r}(s)$ and two orthonormal vectors $\mathbf{d}_{1}$ and $\mathbf{d}_{2}$ which define the position of two orthogonal lines in the cross-section of the rod at *s*. If, by definition,

$$\begin{aligned} \mathbf{d}_{1} = \mathbf{d}_{2} \times \mathbf{d}_{3}, \end{aligned}$$

then the triple $(d_{1}, d_{2}, d_{3})(s)$ defines a “co-moving” rod-centred coordinate system called the *directors* of the rod. If shear is present, the first director is not equal to ${\partial }_{s}{} \mathbf{r}(s)$. In what follows below, all vectors will be expanded in the basis of the directors.

The stress in the rod is defined by the vectors:

$$\begin{aligned} \mathbf{n}(s) = n_{1} \mathbf{d}_{1} + n_{2} \mathbf{d}_{2} + n_{3} \mathbf{d}_{3}, \end{aligned}$$

and

$$\begin{aligned} \mathbf{m}(s) = m_{1} \mathbf{d}_{1} + m_{2} \mathbf{d}_{2} + m_{3} \mathbf{d}_{3}, \end{aligned}$$

where $n_{2}$ and $n_{3}$ are components of the shear force, $n_{1}$ is the tension, $m_{2,3}$ are bending moments about the axes parallel to $\mathbf{d}_{2,3}$ and $m_{1}$ is the twisting moment about $\mathbf{d}_{1}$.

The strain in the rod is defined by two vectors:

$$\begin{aligned}&\mathbf{u}(s) = u_{1} \mathbf{d}_{1} + u_{2} \mathbf{d}_{2} + u_{3} \mathbf{d}_{3},\\&\mathbf{v}(s) = v_{1} \mathbf{d}_{1} + v_{2} \mathbf{d}_{2} + v_{3} \mathbf{d}_{3}, \end{aligned}$$

which are defined by the equations:

$$\begin{aligned} {\partial }_{s} \mathbf{d}_{i} = \mathbf{u} \times \mathbf{d}_{i}, \end{aligned}$$

and

$$\begin{aligned} {\partial }_{s} \mathbf{r} = \mathbf{v}. \end{aligned}$$

### Equilibrium Equations

If $\mathbf{f}$ and $\mathbf{l}$ are the external linear densities of the distributed forces and torques, the equilibrium differential equations which are obtained by balancing forces and moments are given by:

1 $$\begin{aligned}&\frac{d \mathbf{n}}{d s} + \mathbf{f} = 0,\end{aligned}$$

2 $$\begin{aligned}&\frac{d \mathbf{m}}{d s} + \mathbf{v} \times \mathbf{n} + \mathbf{l} = 0, \end{aligned}$$

where the derivative on each left-hand side is the total derivative with respect to *s* in the “co-moving” frame. These include the derivatives of the directors with respect to *s*. Below it will be assumed that for each rod $\mathbf{l} = 0$. The force $\mathbf{f}$ includes gravity, given by $-q \mathbf{e}_{3}$, as well as the force exerted upon a given rod by all other rods.

In terms of ordinary (not total) derivatives, the equations above can be written as:

3 $$\begin{aligned}&\mathbf{n}^{\prime } = \mathbf{n} \times \mathbf{u} - \mathbf{f},\end{aligned}$$

4 $$\begin{aligned}&\mathbf{m}^{\prime } = \mathbf{m} \times \mathbf{u} + \mathbf{n} \times \mathbf{v}. \end{aligned}$$

### Constitutive Equations

In order to make the system of differential equations for $\mathbf{n}$ and $\mathbf{m}$ closed, it is necessary to add to these the so-called constitutive equations through which $\mathbf{u}$ and $\mathbf{v}$ can be expressed in terms of $\mathbf{n}$ and $\mathbf{m}$. We let $\mathbf{y} = \mathbf{v} - \mathbf{d}_{1}$ and employ here the constitutive relations as described in Champneys et al. (1997):

5 $$\begin{aligned} u_{i}(s) = m_{i}(s) / A_{i}, \;\;\; i = 1, 2, 3,\end{aligned}$$

6 $$\begin{aligned} y_{i}(s) = n_{i}(s) / H_{i}, \;\;\; i = 1, 2, 3, \end{aligned}$$

where $A_{2,3}$ are the principal bending stiffnesses about $\mathbf{d}_{2,3}$, $A_{1}$ is the torsional stiffness, $H_{2,3}$ are the transverse shear stiffnesses and $H_{1}$ is the axial stiffness. It is clear that if the number of rods *N* is larger than 1, all the stiffnesses have to acquire an additional index which enumerates the rods, so that e.g, $H^{(n)}_{2}$ is a transverse shear stiffness of the *n*-th rod ($n = 1,2,\ldots , N$).

Furthermore, the force density of interaction between various rods needs to be specified. We therefore let $\mathbf{f} = \mathbf{f}_{j}$ be the linear density of force which acts on the *j*-th rod and assume that it contains two parts,

$$\begin{aligned} \mathbf{f}_{j} = \mathbf{g}_{j} - q \mathbf{e}_{3}, \end{aligned}$$

and $\mathbf{g}_{j}$ is the force exerted on the *j*-th rod by its neighbours. The latter is assumed to be linear:

$$\begin{aligned} g_{j, i} = -k_{j, i} (2 r_{j, i} - r_{j+1, i} - r_{j-1, i}), \end{aligned}$$

where the index *i* denotes the *i*-th component of the force $\mathbf{g}_{j}$ in the “laboratory” frame. When solving the system of differential equations for forces and torques, the above expression for $g_{j, i}$ has to be transformed to the “co-moving” frame for each rod separately.

### Boundary Conditions

The following boundary conditions are assumed:

- Each rod is “clamped” at $s = 0$, that is $\mathbf{r}^{(n)}(0) = 0$ for every $n = 1, 2, \ldots , N$ where *N* is the number of rods. This gives 3*N* conditions.
- For each rod, the moments at the end points ($s = L$) vanish, $\mathbf{m}^{(n)}(L) = 0$, $n = 1, 2, \ldots , N$; this gives a further 3*N* conditions.
- For each rod, the following initial conditions for the directors are assumed:
  $$\begin{aligned}&\mathbf{d}_{1}^{(n)}(0) = \left( \cos (\phi _{n}), \sin (\phi _{n}), 0 \right) \\&\mathbf{d}_{2}^{(n)}(0) = \left( -\sin (\phi _{n}), \cos (\phi _{n}), 0 \right) \\&\mathbf{d}_{3}^{(n)}(0) = \left( 0, 0, 1 \right) \end{aligned}$$
  The equations above form a further 9*N* boundary conditions. The angles $\phi _{n}$ have been taken to be $-\pi /3 + 2 \pi n / (3N)$.
- Since it is assumed that all rods meet at the tip of the leaf, it is further assumed that the coordinates of the end of each rod (in the “laboratory” frame) are the same, that is:
  $$\begin{aligned} x^{(n)}(L) = x^{(n+1)}(L), \;\; y^{(n)}(L) = y^{(n+1)}(L), \;\; z^{(n)}(L) = z^{(n+1)}(L), \end{aligned}$$
  $n = 1, 2, \ldots , N-1$, where $x^{(n)}(L)$, $y^{(n)}(L)$, $z^{(n)}(L)$ are the Cartesian coordinates of the ends of rods in the “laboratory” frame. The equations above form $3N - 3$ boundary conditions.
- Finally, the three components of the total force which act on the tip of the leaf are specified. In particular, if there are no external forces, the tip is “free” so that the total force at $s = L$ is equal to zero. This requirement provides the remaining 3 boundary conditions.
  Thus, a system of 18*N* ordinary differential equations with 18*N* boundary conditions needs to be solved. The symbols used to define the mathematical model together with their dimensionless counterparts have been gathered in Table 1.

**Table 1:** List of symbols used in the description of the model and their meaning

Symbol	Meaning
*L*	Length of any rod of which the model leaf is made
*s*	Arc-length parameter
$\mathbf{r}$	Position vector in the fixed basis
$\mathbf{e}_{i}$	Unit vectors of fixed right-hand basis
$\mathbf{d}_{i}$	Directors - rod-centred coordinate system unit vectors
$n_{1}$	Tension
$n_{2,3}$	Two components of the shear force
$m_{1}$	Twisting moment about the axis parallel to $\mathbf{d}_{1}$
$m_{2,3}$	Two components of bending moments about the axes parallel to $\mathbf{d}_{2}$ and $\mathbf{d}_{3}$
$\mathbf{u}$	Strain vector (defined in terms of the directors)
$\mathbf{v}$	Strain vector (defined in terms of $\mathbf{r}$)
$\mathbf{f}$	Linear density of external distributed forces
$\mathbf{l}$	Linear density of external distributed torques
q	Linear density of gravity force
$\mathbf{y} = \mathbf{v} - \mathbf{d}_{1}$	Deflection of the first director from the tangent line to any rod
$A_{1}$	Torsional stiffness
$A_{2,3}$	Principal bending stiffnesses
$H_{1}$	Axial stiffness
$H_{2,3}$	Transverse shear stiffnesses
$\mathbf{g}_{j}$	Force density exerted on the *j*-th rode by its neighbours
$L_{0}$	Typical length of rods of which the model leaf is made
$\tau = s/L_{0}$	Dimensionless arc-length parameter
(*x*, *y*, *z*)	Cartesian components of the centre-line of any rod
$(\xi , \eta , \zeta ) = (x, y, z)/L_{0}$	Rescaled components of centre-lines of the rods
$H_{0}$	Typical transverse shear stiffness
${\bar{n}}_{k} = n_{k}/H_{0}$	Dimensionless forces
$h_{k}$ = $H_{k}/ H_{0}$	Dimensionless stiffnesses
$A_{0}$	Typical bending stiffness
${\bar{m}}_{k} = (L_{0}/A_{0}) m_{k}$	Dimensionless torques
$a_{k} = A_{k}/A_{0}$	Dimensionless stiffnesses
$\kappa _{k}$	Strength of the coupling between neighbouring rods
${\bar{\kappa }}_k = (L_{0}/H_{0}) \kappa _{k}$	Dimensionless coupling strength between neighbouring rods

It is legitimate to ask whether it would not be sufficient to employ a simpler, linear model, either for each rod, or indeed for the whole leaf. For instance, the Kirchhoff or Timoshenko models of rods in Elishakoff (2020) can be considered. Or, one could consider leaves to be modelled by plates of anisotropic materials (linear or non-linear, Mansfield (1989)). Our choice for Cosserat rod theory is considered the most satisfactory rod theory from a mathematical point of view. It is called “geometrically exact” as it provides both the position of each point of the rod and its orientation. We wanted to take into account that not only bending and twisting, but also stretching (and shearing) of the leaves and hence the modelling rods may appear. The modelling of leaves with the help of plate theories is, of course, very valuable as shown in Liang and Mahadevan (2009), but we have attempted to introduce a model with, on the one hand, great flexibility and on the other hand, which is particularly well suited for strongly anisotropic systems.

## Materials and Methods

### Plant Material

A tensile test was performed on a sample of 72 fresh leaves from orchids belonging to the *Epipactis* genus, i.e. *Epipactis helleborine* (L.) Crantz, *Epipactis albensis* Nováková et Rydlo and *Epipactis palustris* (L.) Crantz were collected from their natural habitat in July 2016 at six different locations in Poland. All the specimens were identified using their morphological character, especially the gynostemium structures, by A.J-B. Five individuals per population were taken for all the experimental analyses and only undamaged leaves were used for the experiment. The selection of leaves for analyses/testing from various species of the genus *Epipactis* was dictated by the fact that the species grow in various habitat conditions. However, the type of plant habitat, elevation, exposition and soil types/conditions determine the leaf structure (both its morphology and anatomy). In order to protect against water loss, the material was transported in bags with moist material and whole shoots of plants were harvested (frames) as in Zwieniecki et al. (2007). The genus *Epipactis* is a clonal orchid, i.e. the rhizome grows underground and produces shoots/ramets of various shapes and sizes of leaf depending on their age and position/location on the shoot. In order to evaluate the change in shape, various leaves within one specimen were studied. Whole leaves were introduced into the pneumatic grips of an Instron machine. Based on the detailed and extensive knowledge of the structure and development of orchid leaves (see, e.g., Arditti (1992) and Jakubska-Busse et al. (2017)), the whole leaves were prepared in such a way that the main thickest venation was preserved. Data on the morphology and anatomy of the *Epipactis* spp. leaves, which had been previously published in Jakubska-Busse and Gola (2010), Jakubska-Busse et al. (2012), and Jakubska-Busse and Gola (2014), was used. The experimental studies and material sampling were done with the permission of the Regional Director for Environmental Protection, Nos.: WPN.6205.123.2016.IL, WPN.6400.32.2016.IL and WPN.6400.33.2016.IL.

### Experimental Procedure

The experimental part of this study was performed in the laboratory at the Meat Technology Division WULS-SGGW (Warsaw, Poland). Leaves blades of *Epipactis* spp. were stretched in both length and width until they were broken off between the grips using the Zwick 1445 Universal Testing System for stretching soft materials. A broad range of mechanical testing can be performed by the laboratory’s testing machine as long as the tests concerned are quasi-static. These strength tests were carried out immediately after the material had been delivered. The experiment lasted 4 hours and was repeated with new fresh material after two weeks. Whole leaves were introduced into the pneumatic grips of an Instron machine. The jaws of the Instron machine were set at 10 and 15 mm, and the leaves were stretched at a rate of 20 mm/min. On the basis of the experimental results obtained, the values of the Young’s modulus were calculated and plotted. Twenty eight important quasi-static stretching tests were done on the leaves. A Python program was used in order to produce results from the model.

### Model Description

In order to obtain the values of the Young’s modulus, the standard definition of the stress as the ratio of the applied force and the cross-section was used. Similarly, the strain was defined as the relative change in the leaf length under the action of the force.

## Experimental Results and Their Analysis

From the experimental data obtained, the Young’s modulus of the leaves were calculated.

**Fig. 1:**
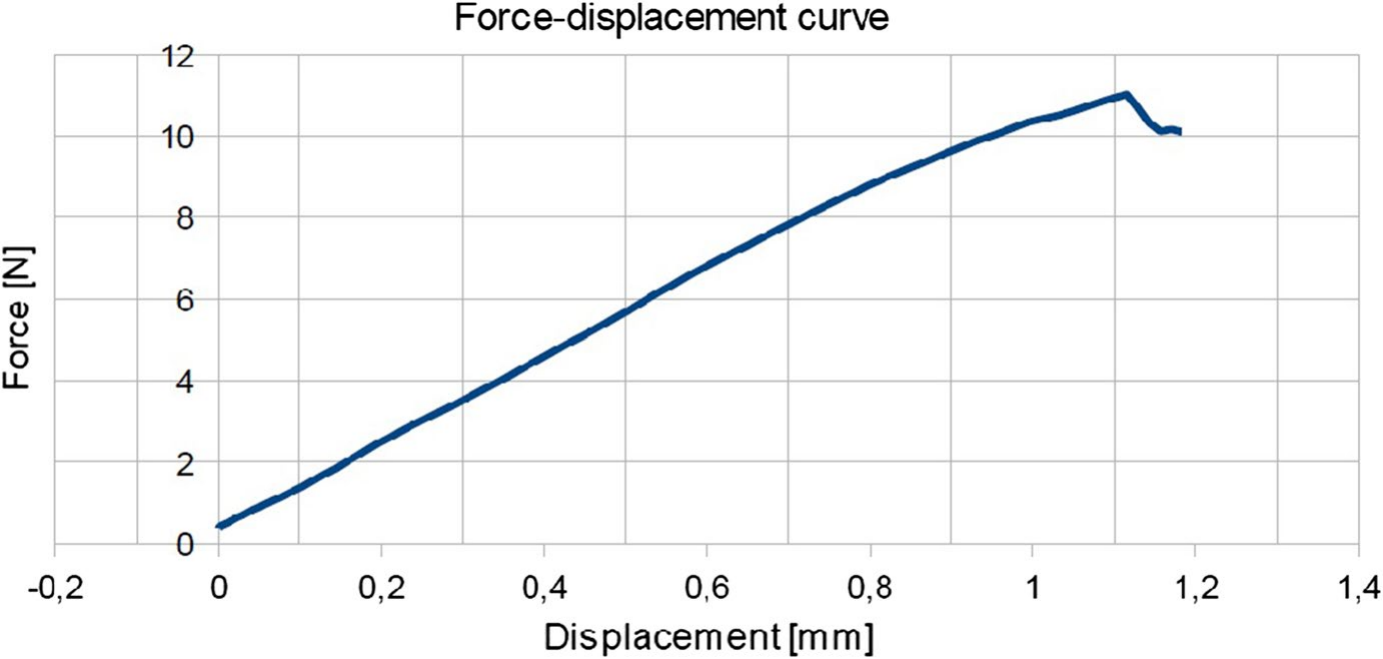
The force-displacement curve for *Epipactis* leaf No. 1 which is given in Table 2. The leaf was stretched in the longitudinal direction of the thickest innervation/venation. This figure has been obtained directly from the measuring device in the Zwick 1445 Universal Testing System.

**Fig. 2:**
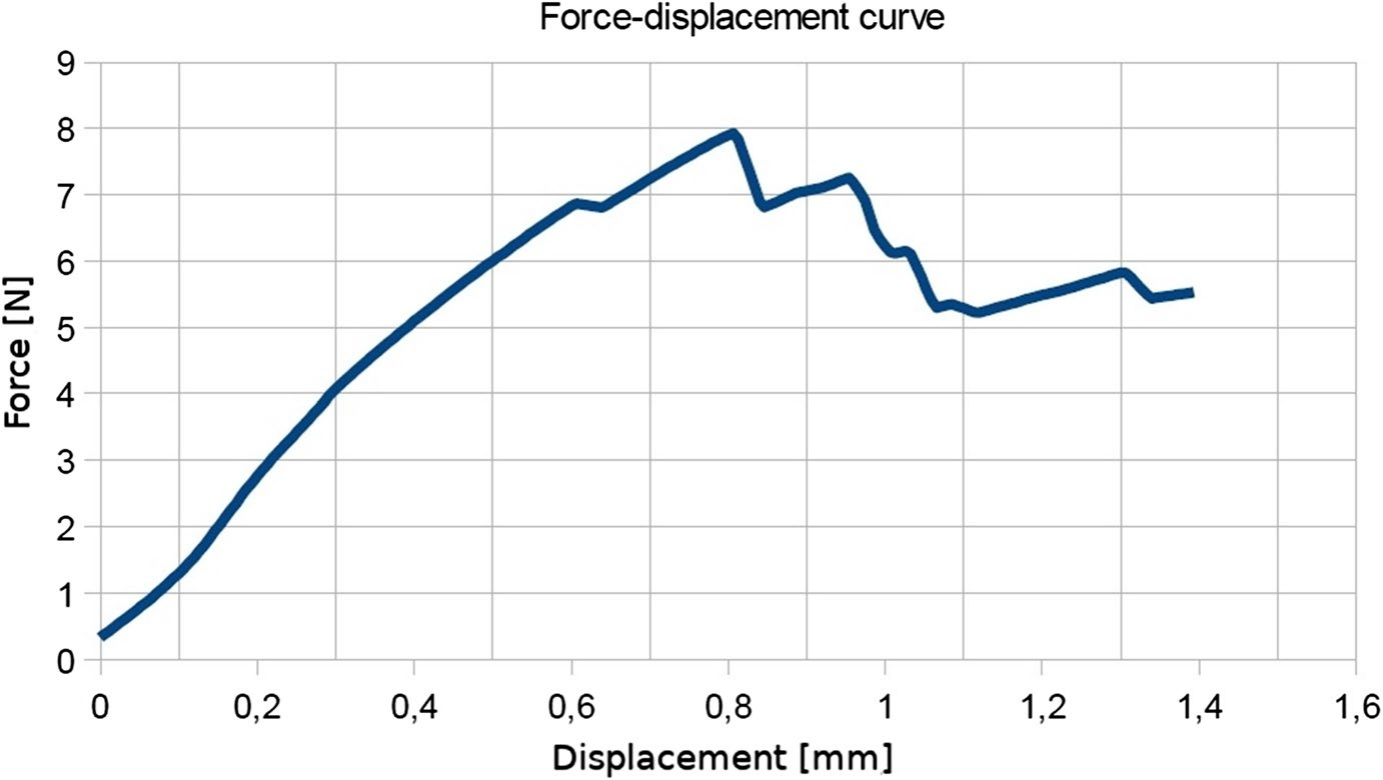
The force-displacement curve for *Epipactis* leaf No. 27 which is given in Table 2. The leaf was tested for tensile strength at a perpendicular orientation relative to the thickest innervation/venation. This figure has been obtained directly from the measuring device in the Zwick 1445 Universal Testing System.

For this, applied force-elongation graphs were plotted (see Fig. 1). The leaf was stretched in the longitudinal direction of the thickest venation. By analysing the curve of Fig. 1, it can be seen that the breaking forces increase linearly with displacement. After reaching the maximum value, there is a mild and irregular decrease until it is almost stable. The process of breaking proceeded gradually and in layers. In another study, some of the authors took into account the morphological and anatomical structure of *Epipactis* species leaves  Jakubska-Busse and Gola (2014). The initial location of breakage in the process of breaking depends on the construction of the leaf layers. Leaves can have similar thickness, but a different number and shape of the cells in the layers. There may be fewer layers in which there are larger cells or more layers with smaller cells. In Fig. 2 before reaching the maximum force, some irregularities can be seen, which can be defined as the initial picking process, and the first micro-breaks appear (small cracks often occurring simultaneously). This process also takes place gradually in layers as in the case shown in Fig. 1. Micro-cracks occurring in the outer layers weaken the leaf structure and the material loses its continuity. It was observed that the leaves were stronger in the longitudinal direction of the thickest innervertion.

**Fig. 3:**
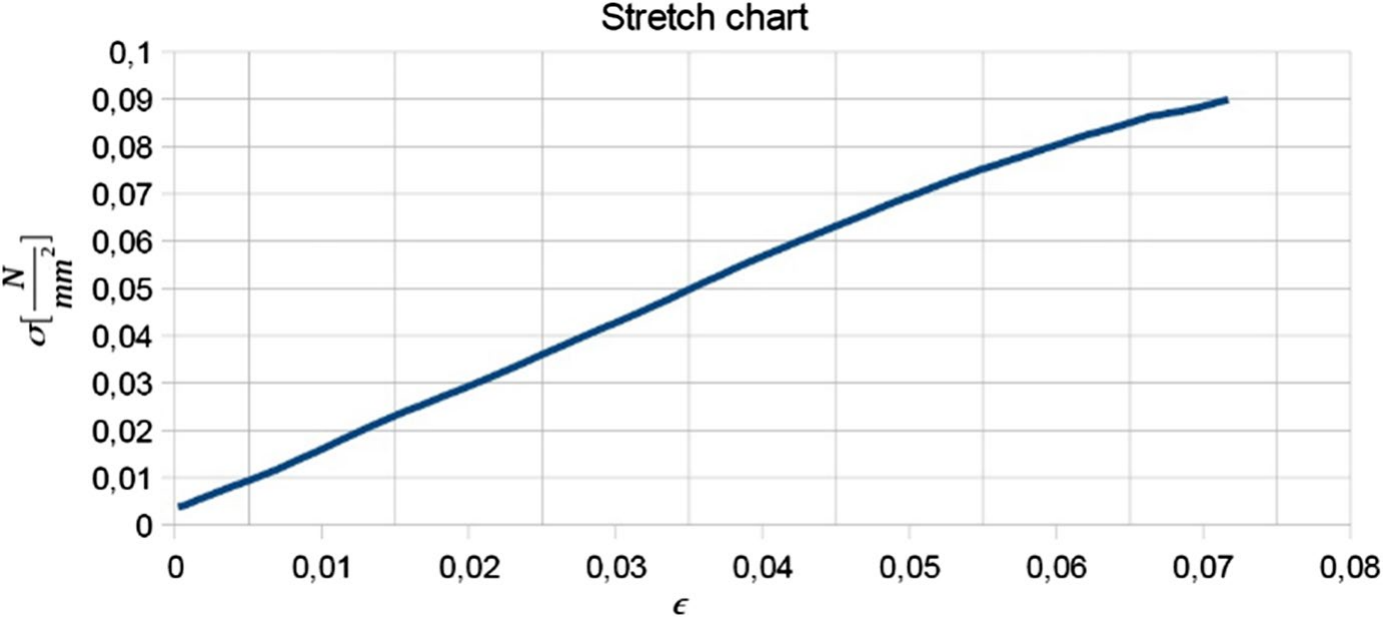
A stress-strain graph for *Epipactis* leaf No. 1 which is given in Table 2

Using the formulae for the stress $\sigma = F/S$ and for the strain $\epsilon = \Delta l / l$ where *F* is the applied force, *S* is the surface of the cross-section of the leaf and *l* is its length in the absence of an external force, stress-strain graphs of the type shown were plotted. The stress-strain relation obtained was linear to a good approximation, such that Hooke’s Law was satisfied, as observed in Fig.  3. From the slope of the best linear fits the Young’s modulus *E* of various leaves were obtained since $\sigma = E \epsilon$.

The values of the Young’s modulus are summarised in Table 2.

**Table 2:** Widths, lengths and Young’s modulus of 27 *Epipactis* leaves measured using the Zwick 1445 Universal Testing System

No.	width [cm]	length [cm]	E [N/mm^2^]
1	6.2	12.0	0.739
2	4.6	10.9	0.494
3	4.2	11.4	0.736
4	2.1	10.2	0.320
5	5.0	13.0	3.747
6	11.0	14.4	0.561
7	4.7	8.0	0.388
8	2.4	7.5	0.433
9	1.6	6.7	0.158
10	2.5	6.8	0.231
11	2.5	9.0	0.349
12	3.2	7.5	0.561
13	3.6	7.3	0.824
14	3.2	12.5	0.534
15	1.8	13.5	0.149
16	1.8	10.7	0.250
17	3.8	14.8	0.326
18	3.5	15.5	0.297
19	4.9	11.2	0.754
20	6.6	16.0	0.803
21	3.3	10.7	0.241
22	2.4	9.7	0.405
23	2.5	7.5	0.187
24	2.2	6.3	0.352
25	2.1	6.6	0.180
26	1.8	6.8	0.197
27	3.4	12.2	0.367

It is clear from Table 2 that the Young’s modulus of the sampled leaves differed quite considerably by up to one order of magnitude although it could be said that typically these are equal to a few tenths of a N/mm$^{2}$. Together with the data on the width and length of a “generic” leaf, this provides useful input for our model. It has been assumed that there is only a single layer of rods which constitute the leaf and in the numerical simulation $\sim 15$ rods were used, which means that the diameter of a rod should be of the order of 0.1 mm. Assuming a circular cross-section for all rods, a value of the order of  10^−6^ N mm^2^ is obtained for the product *EI* of the Young’s modulus and the area moment of inertia.

## Numerical Simulations

The boundary-value problem specified in Sect. 2 was solved numerically with a Python code which used the *solve_bvp* procedure from the *scipy.integrate* package and which is based on an earlier Fortran code developed by Shampine et al. (2006).

It has been convenient to work with dimensionless quantities, hence we let $L_{0}$ be a typical length, equal, for example, to the overall length of the leaf. In terms of $L_{0}$ a dimensionless arclength parameter $\tau = s/L_{0}$ is defined. Similarly, the Cartesian components of the cross-section of any rod (*x*, *y*, *z*) are rescaled to give $(\xi , \eta , \zeta ) = (x, y, z)/L_{0}$. The dimensionless forces ${\bar{n}}^{(m)}_{k}$ ($k=1,2,3$, $m = 1, 2, \ldots , N$) are obtained in terms of a typical transverse shear stiffness $H_{0}$, ${\bar{n}}_{k} = n_{k}/H_{0}$. The resulting dimensionless stiffnesses are denoted by lower case *h*, that is, $h^{(n)}_{k}$ = $H^{(n)}_{k}/ H_{0}$. Finally, the dimensionless torques are obtained by using a typical bending stiffness $A_{0}$, ${\bar{m}}^{(n)}_{k} = (L_{0}/A_{0}) m^{(n)}_{k}$. The dimensionless stiffnesses $a^{(n)}_{k}$ are then defined as $A^{(n)}_{k}/A_{0}$, and the dimensionless couplings ${\bar{\kappa }}_k$ as $(L_{0}/H_{0}) \kappa _{k}$.

**Fig. 4:**
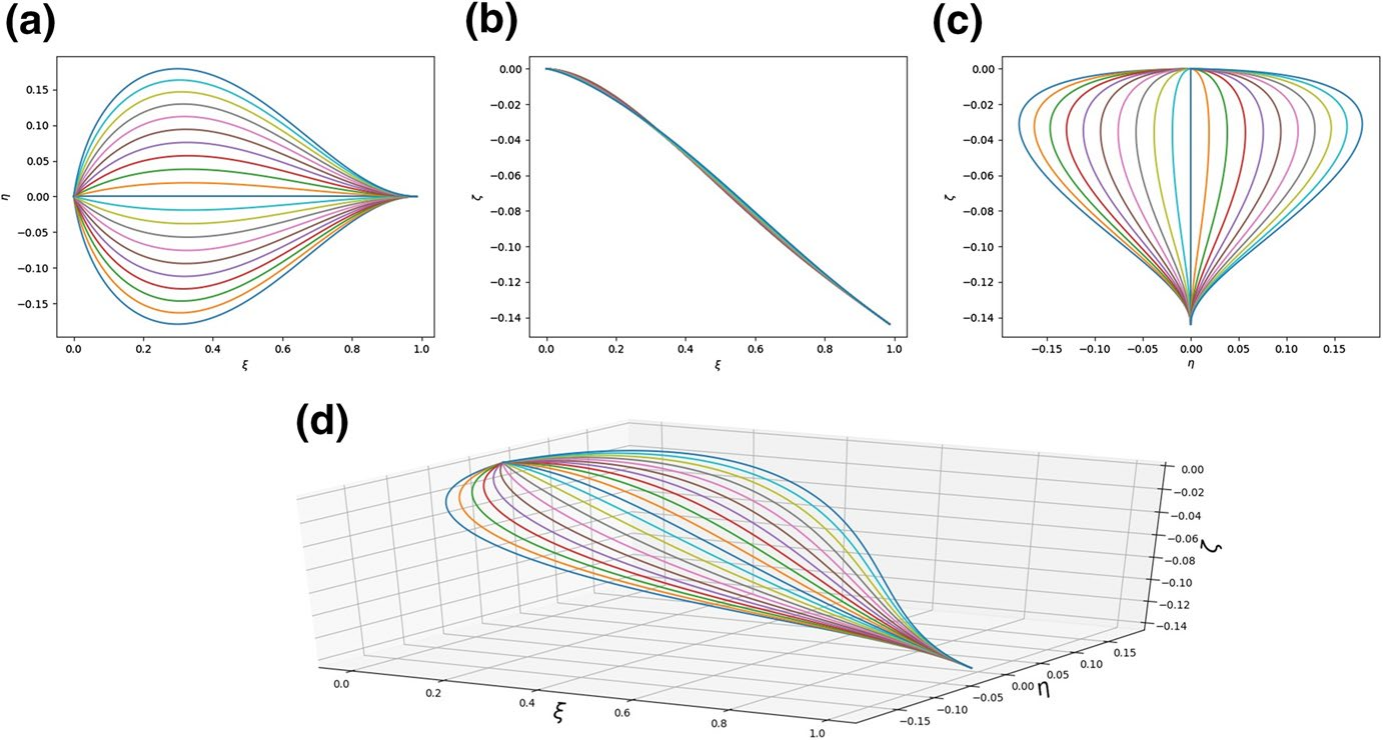
**a** The shape of the system of rods modelling the leaf as seen in the $\xi -\eta$ plane; **b** The shape of the system of rods modelling the leaf as seen in the $\xi -\zeta$ plane; **c** The shape of the system of rods modelling the leaf as seen in the $\eta -\zeta$ plane; **d** A diagram of the system of rods in three dimensions. The parameters are $a^{(n)}_{k} = 1$, $h^{(n)}_{k} = 100.0$ for $n = 1, 2,\ldots , N$, $k = 1, 2, 3$, ${\bar{\kappa }}_{y} = {\bar{\kappa }}_{z} = 1$, $q = 1.0$.

We experimented with several numbers of rods (between 5 to 35) and found that sufficient detail in the figure is obtained for *N* no larger than 20. In particular, to plot Fig. 4 the leaf was simulated with $N = 21$ and the parameters of the system were $a_{i}^{(n)} = h_{i}^{(n)} = 1$ for $i = 1,2,3$ and $n=1,2,\ldots , N$, ${\bar{\kappa }}_{y} = {\bar{\kappa }}_{z} = 1$. Each curve in the figures below represents a single rod which is a model of a vein together with its surrounding tissue. Thus, *N* is the “number of veins”. In fact the real number of veins was not counted. Using the latter number in the simulations would not make the shape of the model leaf more realistic whilst also then making the simulations take an unreasonably long time.

**Fig. 5:**
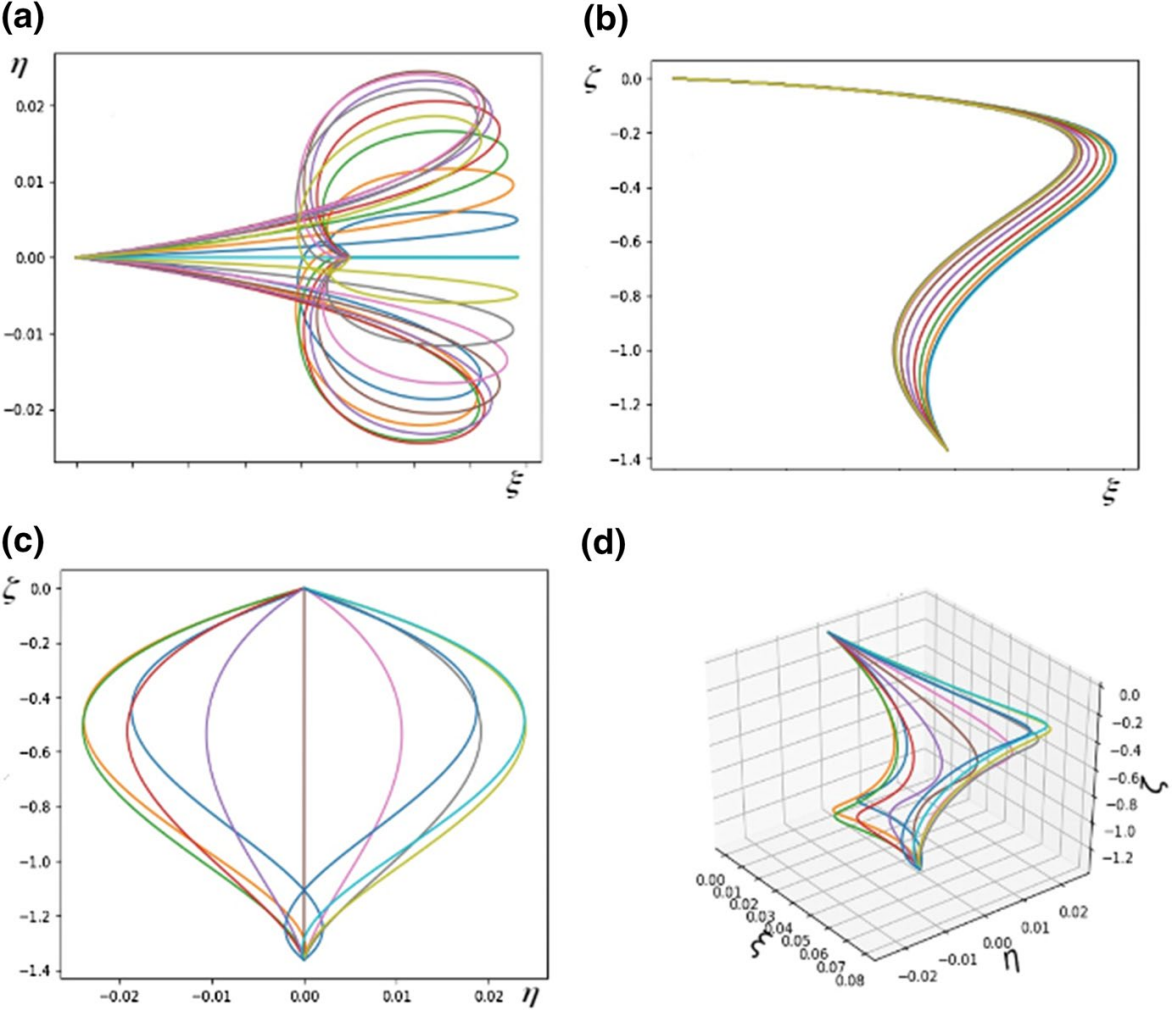
**a** The shape of the system of rods modelling the leaf as seen in the $\xi -\eta$ plane; **b** The shape of the system of rods modelling the leaf as seen in the $\xi -\zeta$ plane; **c** The shape of the system of rods modelling the leaf as seen in the $\eta -\zeta$ plane; **d** A diagram of the system of rods in three dimensions. The parameters are $a^{(n)}_{1} = 20, a^{(n)}_{2} = 20, a^{(n)}_{3} = 0.3, h^{(n)}_{k} = 1.0$, ${\bar{\kappa }}_{y} = {\bar{\kappa }}_{z} = 1$

The richness of possible structures which can be obtained within the model can be seen by a comparison of Figs. 4 and 5 in which different parameter sets were used.

**Fig. 6:**
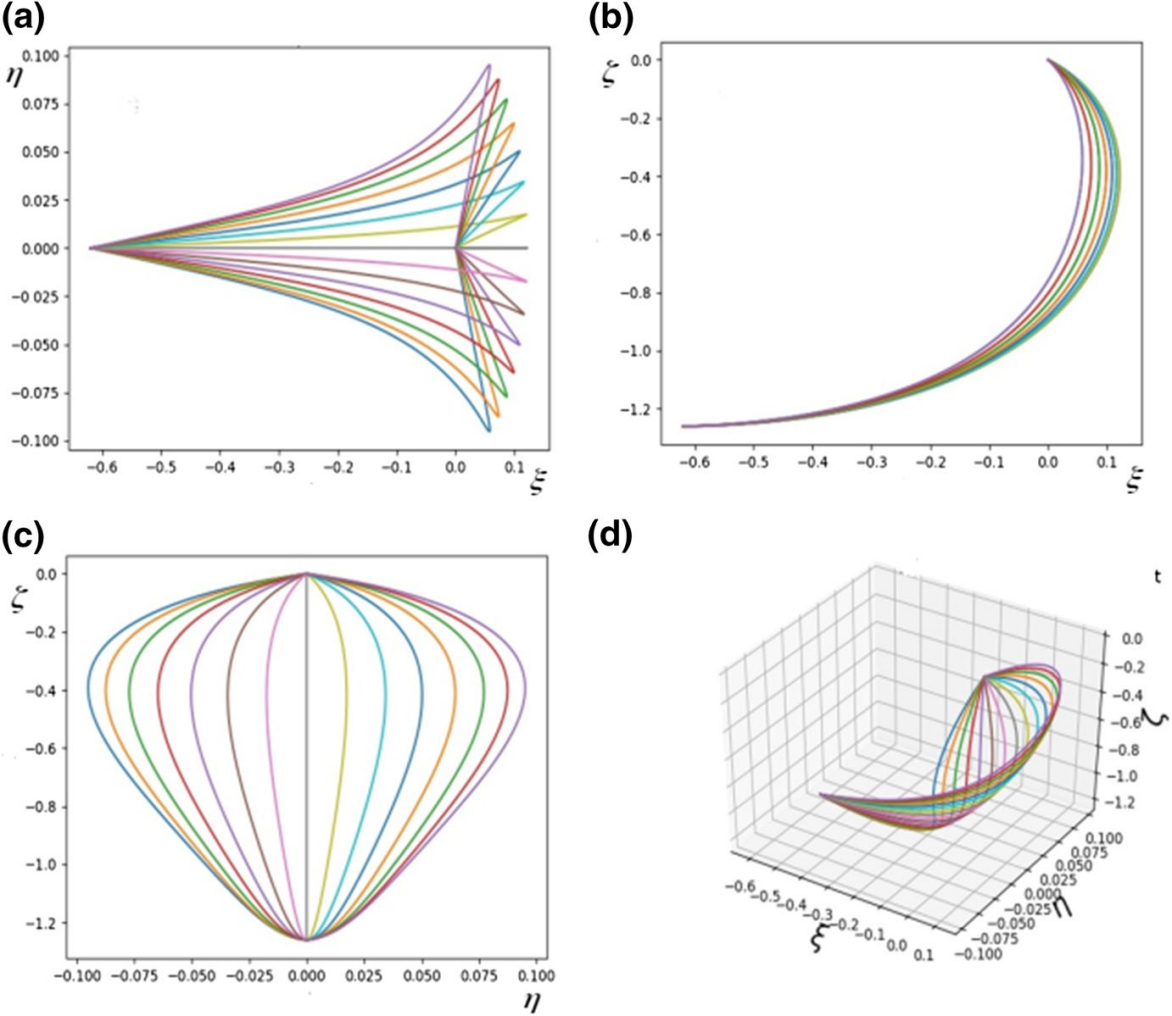
**a** The shape of the system of rods modelling the leaf as seen in the $\xi -\eta$ plane; **b** The shape of the system of rods modelling the leaf as seen in the $\xi -\zeta$ plane; **c** The shape of the system of rods modelling the leaf as seen in the $\eta -\zeta$ plane; **d** A diagram of the system of rods in three dimensions. The parameters are $a^{(n)}_{k} = 1, h^{(n)}_{k} = 10$, $u^{(n)}_{2} = 1.0$, for $n = 1,2, \ldots , N$, $k = 1, 2, 3$

The model can also take into account conditions of non-vanishing initial curvature. “Initial” means here that it also appears in the absence of any external forces or torques. To model this, we write:

$$\begin{aligned} u^{(n)}_{k} = u^{(n)}_{0k} + m^{(n)}_{k} / A^{(n)}_{k}, \end{aligned}$$

and the initial curvatures are characterised by non-vanishing $u^{(n)}_{0k}$. Figure 6 shows the effect of $u^{(n)}_{2} = 1.0$ for $n = 1, 2, \ldots , N$ for $N = 15$.

### Discussion Based on a Linearised Model

In this section we attempt to find some insight into the possible reason or reasons for wrinkling in an elementary way based on a linearisation of the Cosserat model. We consider here only one rod, namely, one of the lateral rods in our rod-based model of the leaf.

Let us start with the relation:

$$\begin{aligned} \mathbf{r}^{\prime } = \mathbf{v} = \mathbf{d}_{1} + \mathbf{y}. \end{aligned}$$

For the components $r_{i}$ of the vector $\mathbf{r}$ we have, therefore:

$$\begin{aligned} r_{i}^{\prime } = d_{1i} + \frac{n_{i}}{H_{i}} \end{aligned}$$

Let us now differentiate again over *s* and neglect the second term (for instance, due to large values of $H_{i}$) to obtain:

$$\begin{aligned} r_{i}^{\prime \prime } \approx \epsilon _{ijk} \frac{m_{j}}{A_{j}} d_{1k} \end{aligned}$$

where $\epsilon _{ijk}$ is a totally antisymmetric symbol and the summation convention has been used. It follows that the second and third components of $\mathbf{r}$, *Y* and *Z*, satisfy the equations:

7 $$\begin{aligned}&Y^{\prime \prime } = \frac{m_{3}}{A_{3}},\end{aligned}$$

8 $$\begin{aligned}&Z^{\prime \prime } = -\frac{m_{2}}{A_{2}}, \end{aligned}$$

which is valid provided that $\mathbf{d}_{1}$ does not differ much from $\mathbf{e}_{x}$.

We proceed with differentiating Eq. (4) over *s* while neglecting the first term. Thus we obtain:

9 $$\begin{aligned} \mathbf{m}^{\prime \prime } = - \mathbf{f} \times \mathbf{v} + \mathbf{n} \times \mathbf{v}^{\prime }. \end{aligned}$$

Now, approximating $\mathbf{v}^{\prime }$ with $\mathbf{d}_{1}^{\prime }$ and using $d_{11}^{\prime } \approx 1$, $d_{12}^{\prime } = Y^{\prime \prime }$, $d_{13}^{\prime } = Z^{\prime \prime }$, we get

10 $$\begin{aligned}&m_{2}^{\prime \prime } \approx -f_{3} + n_{1} Z^{\prime \prime },\end{aligned}$$

11 $$\begin{aligned}&m_{3}^{\prime \prime } \approx f_{2} + n_{x} Y^{\prime \prime }. \end{aligned}$$

Using (7, 8) and renaming $n_{1}$ as *T*, we obtain the well-known fourth-order equation for *Y* and *Z*:

12 $$\begin{aligned} Y^{\prime \prime \prime \prime } \approx \frac{f_{2}}{A_{3}} + \frac{T}{A_{3}} Y^{\prime \prime }, \end{aligned}$$

and

13 $$\begin{aligned} Z^{\prime \prime \prime \prime } \approx \frac{f_{3}}{A_{2}} + \frac{T}{A_{2}} Z^{\prime \prime }. \end{aligned}$$

The latter equations coincide (up to the names of components) with Eqs. (20.14) of Landau and Lifshitz (1993). Following the latter reference, we will consider *T* not as a dynamical variable as in the rigorous Cosserat theory, but rather as a unknown constant force which may be both compressive ($T < 0$) and tensile ($T > 0$).

Now, with $f_{2} = 0$, $f_{3} = q$, the solution is elementary:

$$\begin{aligned}&Y(s) = Y_{0} + Y_{1} s + Y_{2} \exp {(\sqrt{T/A_{3}} s)} + Y_{3} \exp {(-\sqrt{T/A_{3}} s)},\\&Z(s) = Z_{0} + Z_{1} s - \frac{1}{2} \frac{q}{T} s^{2} + Z_{2} \exp {(\sqrt{T/A_{2}} s)} + Z_{3} \exp {(-\sqrt{T/A_{2}} s)}, \end{aligned}$$

where $Y_{i}$, $Z_{i}$, $i = 0, 1, 2, 3$ are constants of integration.

The square roots in the above solutions can of course be negative if the force *T* is compressive. This can lead to the oscillatory behaviour of the solutions provided that $|T|/A_{j} L$ are sufficiently large and the boundary conditions allow for the oscillatory mode to appear. In the case of a single rod, the natural boundary conditions are those below Landau and Lifshitz (1993)

$$\begin{aligned}&Y(0) = 0, \; Y^{\prime }(0) = const, \; Y^{\prime \prime }(L) = 0,\; Y^{\prime \prime \prime }(L) - (T/A_{3}) Y^{\prime } = F_{y0},\\&Z(0) = 0, \; Z^{\prime }(0) = const, \; Z^{\prime \prime }(L) = 0,\; Z^{\prime \prime \prime }(L) - (T/A_{2}) Z^{\prime } = F_{z0}. \end{aligned}$$

which express the assumption that the rod is clamped at $s = 0$ and at $s = L$ the torque vanishes while the force is a non-zero constant (in the case of many rods, the total force acting at the tip must be zero, but the forces acting separately on each rod at the tip may be and often are quite large).

These boundary conditions do not prohibit the presence of the oscillatory mode. It can be particularly pronounced and simulate the long-leaf wrinkling if $|T| L/A_{3} \ll |T| L / A_{2}$ or $A_{2} \ll A_{3}$.

The above linear model is unsatisfactory for many reasons, the most important being that it does not says anything about the dependence of the $x-$components of dynamical vector variables on *s* and in rather uncontrolled ways it neglects non-linearities. However, it predicts that wrinkling can take place provided that the ratio $|T| L / A_{2}$ and constants $Z_{2}, Z_{3}$ are large enough—the condition likely to be met in the case of lateral rods.

## Comparison of the Model with Experiment

**Fig. 7:**
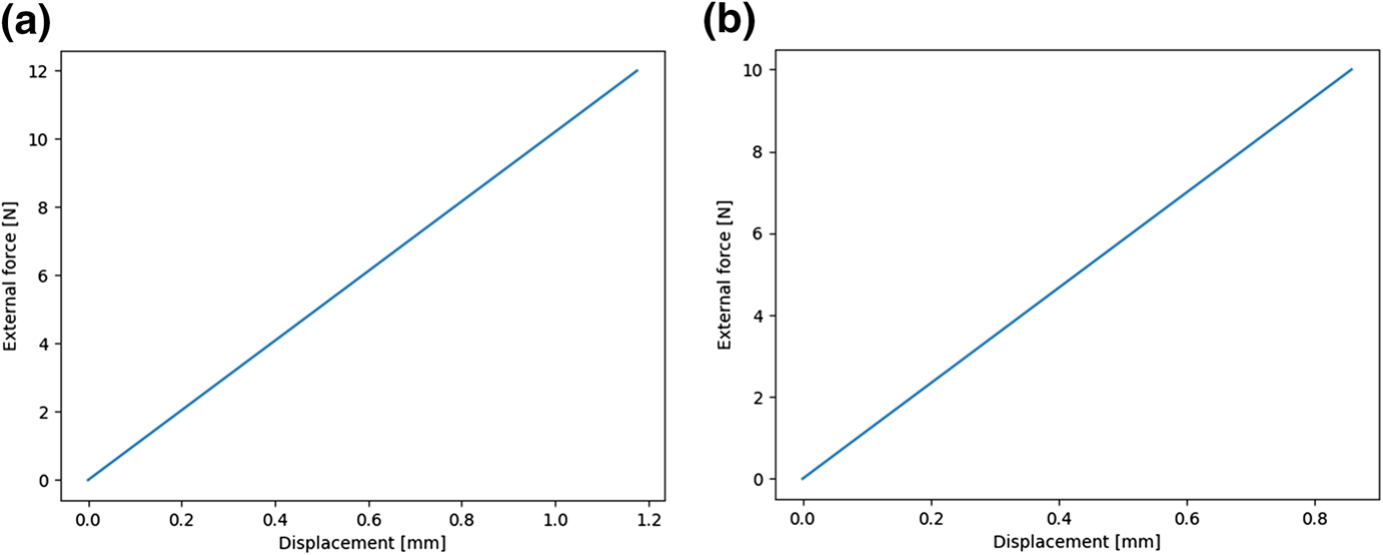
The force-displacement curves obtained from the Cosserat-rods model, cf. Figs. 1 and 2. The parameters are: $N = 21$, $A^{(n)}_{1} = 100\; \text{N cm}^{2}, A^{(n)}_{2} = 150\; \text{N cm}^{2}, A^{(n)}_{3} = 200\; \text{N cm}^{2}$, $H^{(n)}_{1} = 9\; \text{N}, H^{(n)}_{2} = 5\; \text{N}, H^{(n)}_{3} = 0.2\; \text{N}$ in **a** and $A^{(n)}_{1} = 100\; \text{N cm}^{2}, A^{(n)}_{2} = 150\; \text{N cm}^{2}, A^{(n)}_{3} = 200\; \text{N cm}^{2}$, $H^{(n)}_{1} = 10.29\; \text{N}, H^{(n)}_{2} = 5\; \text{N}, H^{(n)}_{3} = 0.2\; \text{N}$ in **b**. The linear density of gravity *q* was equal to $10^{-3}$ N/cm, and the coupling constants have been set to zero

**Fig. 8:**
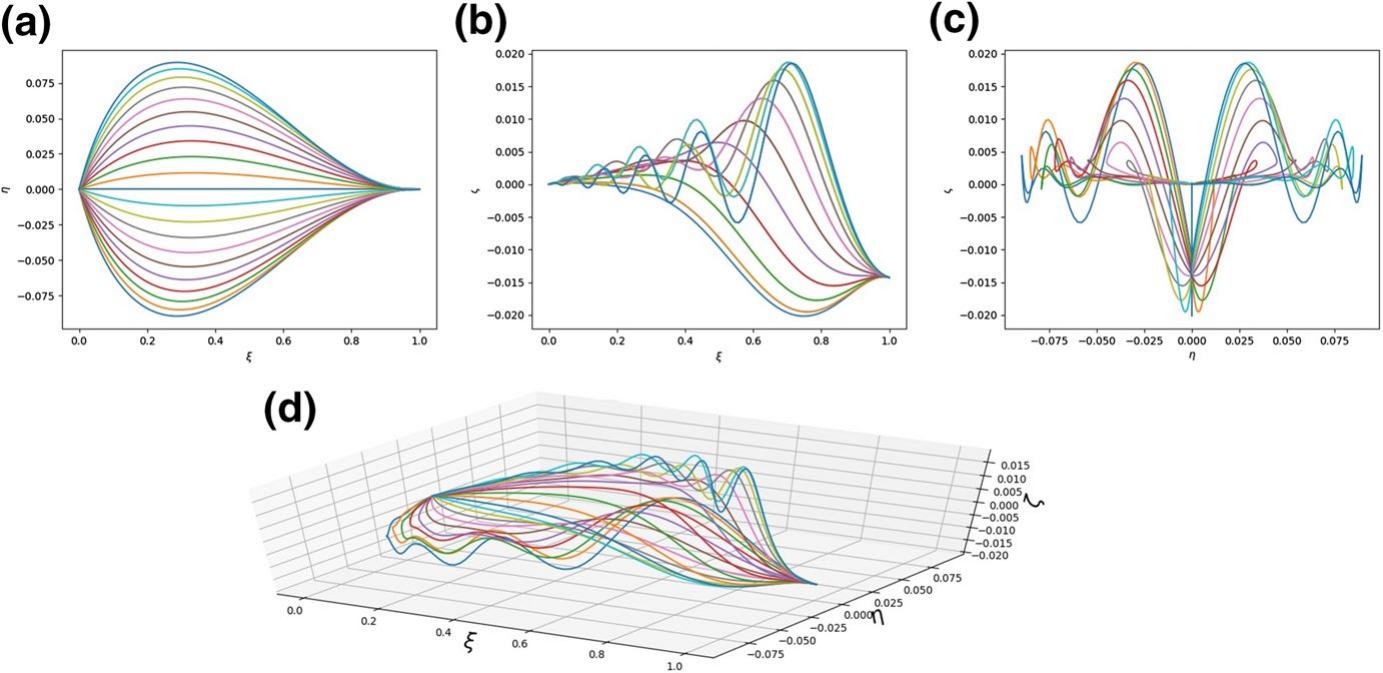
**a** The shape of the system of rods modelling the leaf as seen in the $\xi -\eta$ plane; **b** The shape of the system of rods modelling the leaf as seen in the $\xi -\zeta$ plane; **c** The shape of the system of rods modelling the leaf as seen in the $\eta -\zeta$ plane; **d** A diagram of the system of rods in three dimensions. The parameters are $a^{(n)}_{1} = a^{(n)}_{3} = 1.0$, $a^{(n)}_{2} = 2.5 \cdot 10^{-4}$, $h^{(n)}_{k} = 3$ for $n = 1, 2,\ldots , N$, $k = 1, 2, 3$, ${\bar{\kappa }}_{y} = {\bar{\kappa }}_{z} = 1$, $q = 0.002$

**Fig. 9:**
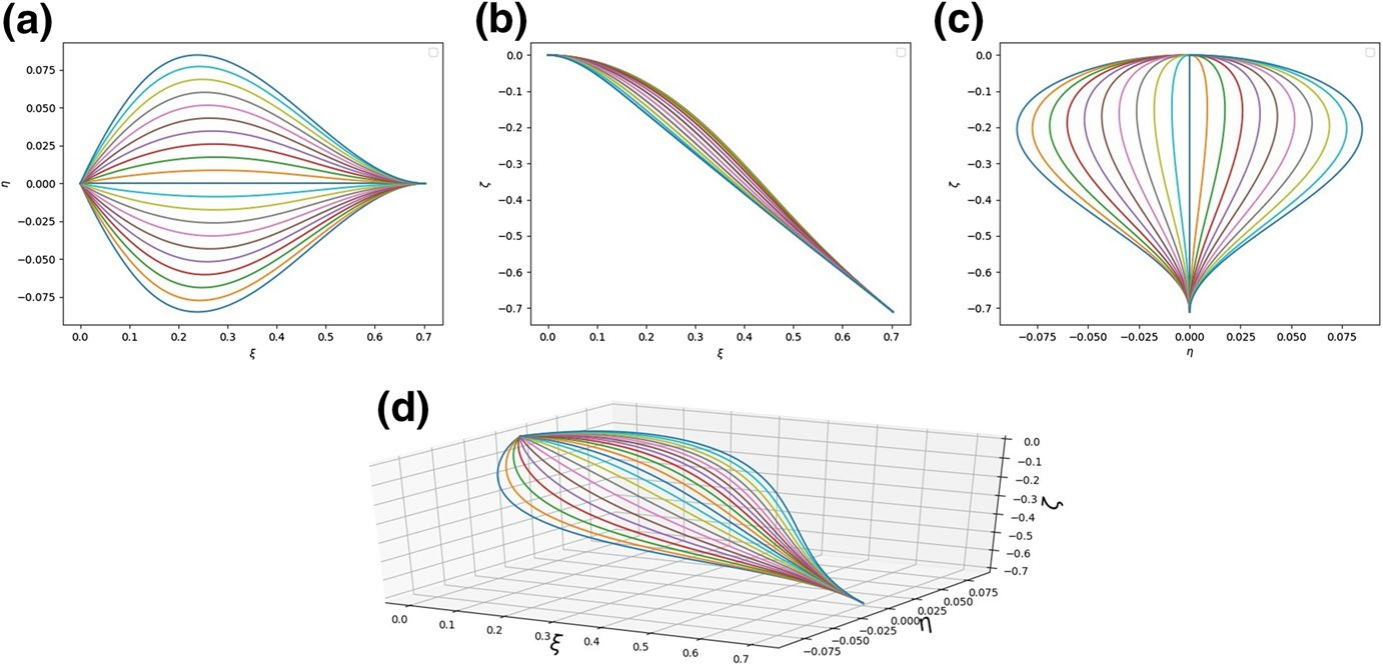
**a** The shape of the system of rods modelling the leaf as seen in the $\xi -\eta$ plane; **b** The shape of the system of rods modelling the leaf as seen in the $\xi -\zeta$ plane; **c** The shape of the system of rods modelling the leaf as seen in the $\eta -\zeta$ plane; **d** A diagram of the system of rods in three dimensions. The parameters are $a^{(n)}_{1} = 1.0$, $a^{(n)}_{2} = 2 \cdot 10^{-4}$, $a^{(n)}_{3} = \cdot 10^{-2}$, $h^{(n)}_{k} = 3$ for $n = 1, 2,\ldots , N$, $k = 1, 2, 3$, ${\bar{\kappa }}_{y} = {\bar{\kappa }}_{z} = 1$, $q = 0.002$.

Let us immediately note that experiments such as ours can neither vindicate nor refute the model as the latter is inherently multi-parameter and it is not very difficult to find parameters $H_{i}$ and $A_{i}$ such that the experimental force-displacement diagrams can be reproduced. In fact, in Fig. 7 it can be seen that the diagrams based on numerical simulations correspond very well to those of Figs. 1 and 2 in the regions of forces where the breaking of the leaf structure took place:

We can also attempt to validate our model qualitatively by checking whether it is able to exhibit the characteristic undulation. To do this, it is reasonable to take into account the analysis of the linearised model in the previous section. It predicts that undulation can take place for relatively small values of the coefficient $A_{2}$. The following figure confirms that expectation (numerical results have been obtained from the full, not linearised, model) (Fig. 8):

However, as demonstrated in Fig. 9, it is not really the smallness of $A_{2}$ per se which may be responsible for the leaf wrinkles, but rather the mismatch between two bending stiffnesses. If indeed $a_{2} \ll a_{3}$, it may be energetically preferable for the lateral rods to form vertical waves rather than to expand to increase the width of the leaf. In Fig. 9 the two stiffnesses are comparable and no undulations are visible.

We would again like to stress that because our model inherently contains many parameters, it would be wrong to assess that it has been confirmed by the experiments in any sense. We can only claim that it has not been invalidated by them.

**Fig. 10:**
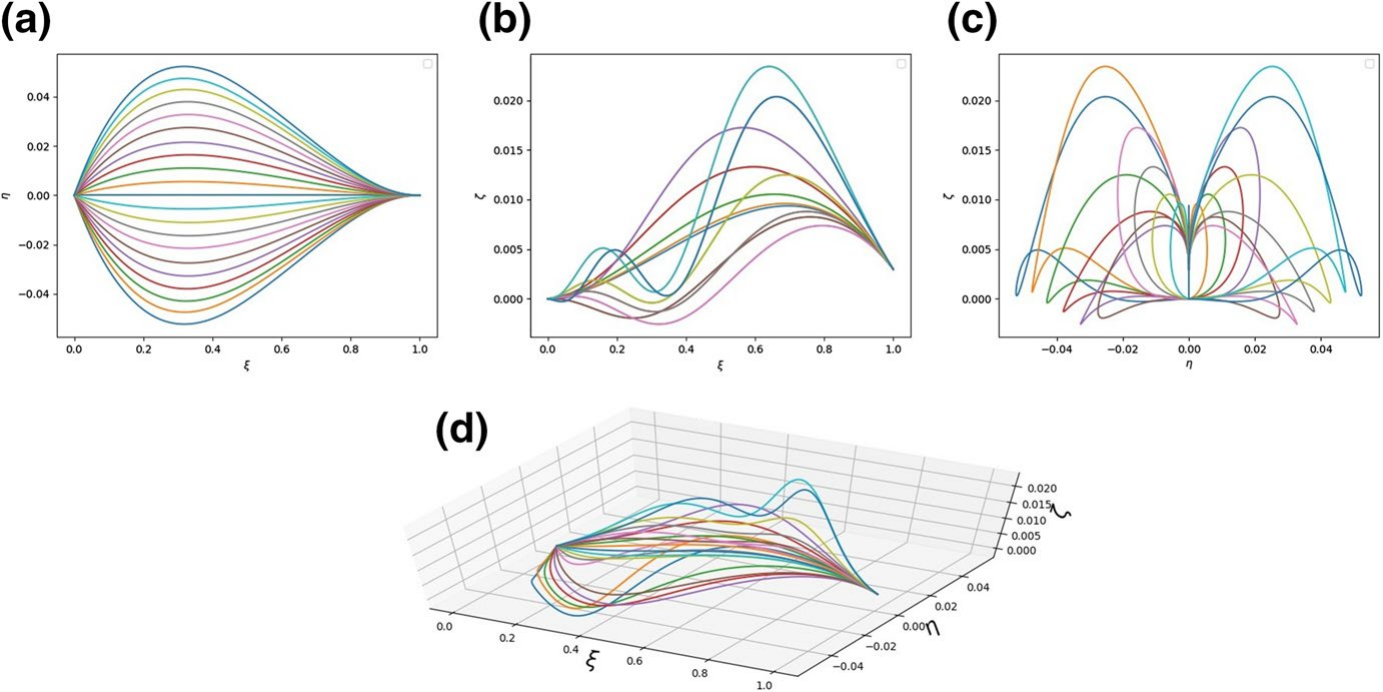
**a** The shape of the system of rods modelling the leaf as seen in the $\xi -\eta$ plane; **b** The shape of the system of rods modelling the leaf as seen in the $\xi -\zeta$ plane; **c** The shape of the system of rods modelling the leaf as seen in the $\eta -\zeta$ plane; **d** A diagram of the system of rods in three dimensions. The parameters are $a^{(n)}_{1} = 8 \times 10^{-3}$, $a^{(n)}_{2} = \times 10^{-1}$, $a^{(n)}_{2} = 1$, $h^{(n)}_{k} = 3$ for $n = 1, 2,\ldots , N$, $k = 1, 2, 3$, ${\bar{\kappa }}_{y} = {\bar{\kappa }}_{z} = 1$, $q = 0.002$

We have also found that relatively small torsional stiffnesses $a_{1}$ reduce the mismatch between the $a_{2}$ and $a_{3}$ (with $a_{2}$ being small) needed for wavy structures to appear. This is exemplified in Fig. 10.

In our numerical simulations we have not taken into account that the lateral parts of the leaf have smaller stiffnesses than its central parts. In fact, the cross-sections of *Epipactis* leaves are by no means homogeneous, please see, e.g., Fig. 4 in Jakubska-Busse and Gola (2010) and Fig. 3 in Jakubska-Busse et al. (2012). These cross-sections include regions which are porous, but the porosity is rather different from that of the aquatic macrophytes investigated in Zhao (2015). This is mainly due to the significant differences in the anatomical structure of leaves between aquatic plants, especially of the heterophyllous species (taxa capable of producing different types of leaves below and above water), and terrestrial orchids representing by the genus *Epipactis*. The inhomogeneity and porosity can of course be taken into account by our model, but we have decided not to do this in order to avoid a proliferation of the (already considerable) number of parameters, the values of which are difficult to estimate.

**Fig. 11:**
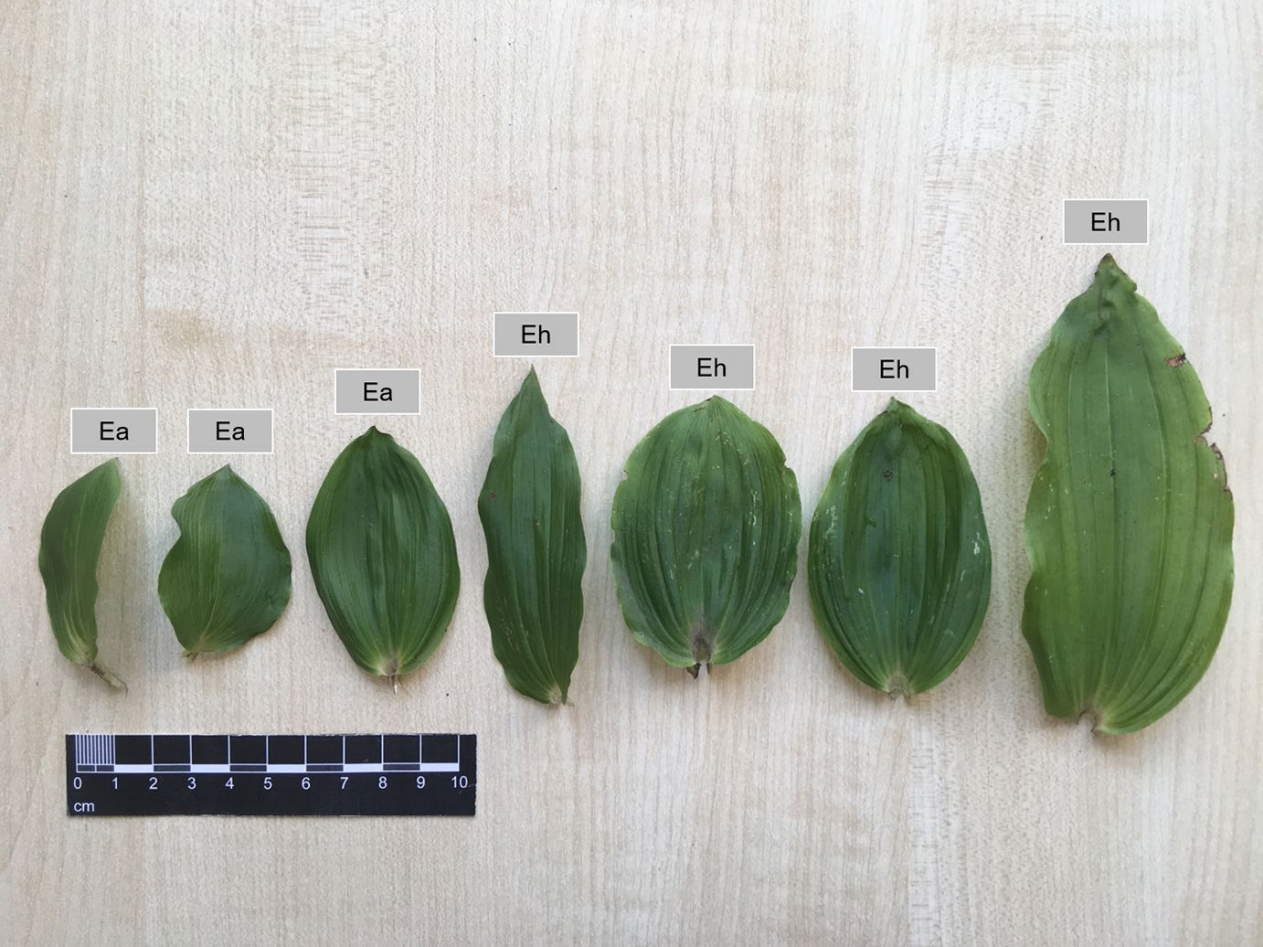
Variation in leaf shape of the investigated plants of genus *Epipactis*. Ea—*Epipactis albensis*, Eh—*Epipactis helleborine*

**Fig. 12:**
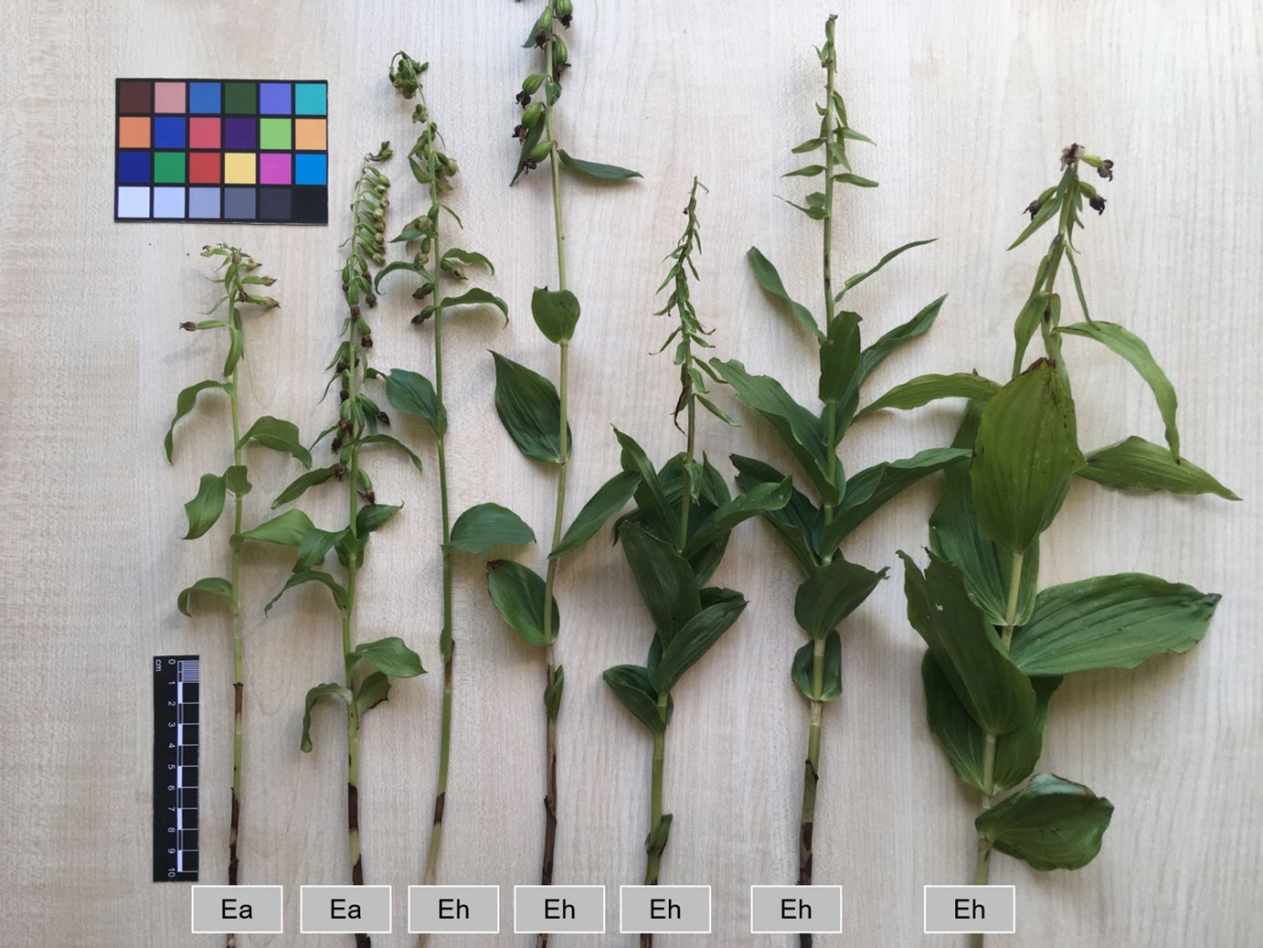
The habit of whole plants of some investigated *Epipactis *species. Ea—*Epipactis albensis*, Eh—*Epipactis helleborine*

Shapes of the real leaves of *Epipactis* are shown in Fig. 11 while Fig. 12 presents the whole fresh plants.

**Fig. 13:**
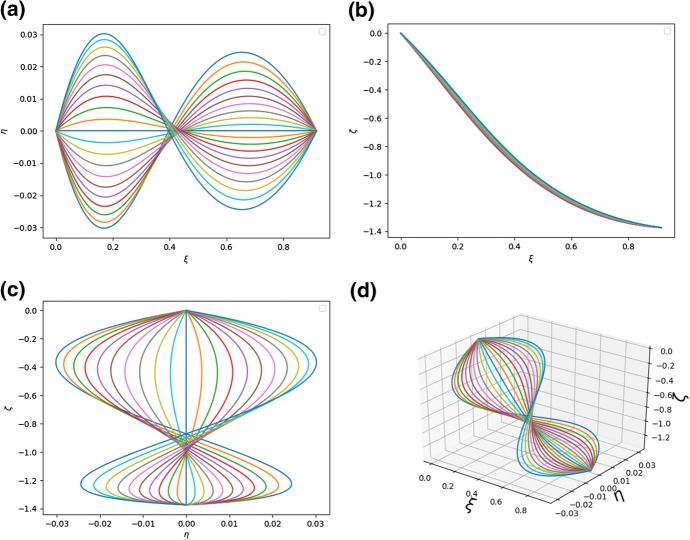
**a** The shape of the system of rods modelling the leaf as seen in the $\xi -\eta$ plane; **b** The shape of the system of rods modelling the leaf as seen in the $\xi -\zeta$ plane; **c** The shape of the system of rods modelling the leaf as seen in the $\eta -\zeta$ plane; **d** A diagram of the system of rods in three dimensions. The parameters are $a^{(n)}_{1} = a^{(n)}_{2} = a^{(n)}_{3} = 1$, $h^{(n)}_{1} = h^{(n)}_{2} = 1$, $a^{(n)}_{3} = 0.4$ for $n = 1, 2,\ldots , N$, ${\bar{\kappa }}_{y} = {\bar{\kappa }}_{z} = 1$, $q = 1$

To demonstrate flexibility of our model let us finally produce pictures which show that it can take into accounting twisting chirality of leaves (or other plant organs) Fig. 13.

## Conclusion

This study considered long leaves with parallel venation as exemplified by the leaves of the genus *Epipactis*. In the experimental part of our study, the extensions of the leaves as functions of applied forces were measured and it was found that Hooke’s Law serves as a very good approximation for those functions. Approximate values of the Young’s modulus of the leaves were determined. In the theoretical part of this study, long leaves with parallel venation were considered as systems of quasi-parallel Cosserat rods coupled with themselves through linear forces and influenced by gravity.

We have demonstrated that this model can take into account the initial curvatures of (a part of) the leaf as well as external tensile forces. In contrast to our previous study, we have posited that the wrinkling observed in many long leaves can be attributed to the mismatch of their bending stiffnesses. Furthermore, small torsional stiffness can enhance the wrinkling in the sense that this can appear when there is a smaller mismatch of the bending stiffnesses.

The determination of the shapes of rods which constitute the model is computationally intensive even with the use of the *solve_bvp* module. In particular, difficulties were encountered in maintaining the three directors orthogonal to each other. The matrix to switch between the “laboratory” and “co-moving” bases occasionally turned out to be ill-conditioned.

Further progress in the understanding of the mechanical structure of leaves can be achieved, firstly through very careful and detailed measurements and also by the use of ab initio “bottom-up” models which start at the level of individual cells.

We believe that the model of coupled rods developed here could be used to describe the qualitative features of other anisotropic organic tissues and possibly also other anisotropic materials.
